# 2741. Hit Me with Your Best Shot: Improving Pre-Lung Transplant Vaccination Rates with Infectious Disease Evaluation

**DOI:** 10.1093/ofid/ofad500.2352

**Published:** 2023-11-27

**Authors:** Gustavo Contreras, Marisa Holubar, Sa Shen, Gundeep Dhillon, Zeynep Tulu, Joanna K Nelson

**Affiliations:** Stanford University School of Medicine, Stanford, California; Stanford University School of Medicine, Stanford, California; Quantitative Sciences Unit, Stanford, California; Stanford University School of Medicine, Stanford, California; Stanford Healthcare, Stanford, California; Stanford University, Stanford, CA

## Abstract

**Background:**

Vaccine preventable diseases and reactivation of latent infections occur commonly post-solid organ transplant and can result in severe infections. Vaccination and screening for latent infections in the pre-transplant period are important tools to mitigate this risk. Previous studies have shown that, despite guidelines, vaccine uptake in the pre-transplant population is suboptimal.

**Methods:**

We performed a quality improvement project in which all patients >=18 years old being evaluated for lung transplant (LT) were referred for Infectious diseases (ID) evaluation with the endorsement of our LT Quality Council starting in 11/2021. ID evaluation included screening for and treating latent infections, vaccination history and drug allergy review, adjusting peri-operative antibiotics, and patient education. To assess the impact of our intervention, we conducted a retrospective chart review and extracted demographics, transplant status, and recommended and completed vaccinations. We compared vaccination adherence of consecutive patients who underwent ID evaluation (11/2021 – 8/2022) to a historical cohort of patients who were not evaluated by ID but underwent LT (1/2019 – 12/2020). We defined adherence for each vaccine (Table 1) and compared the odds of vaccination between groups using odds ratios. This project was deemed non-human subject research.

**Figure:**
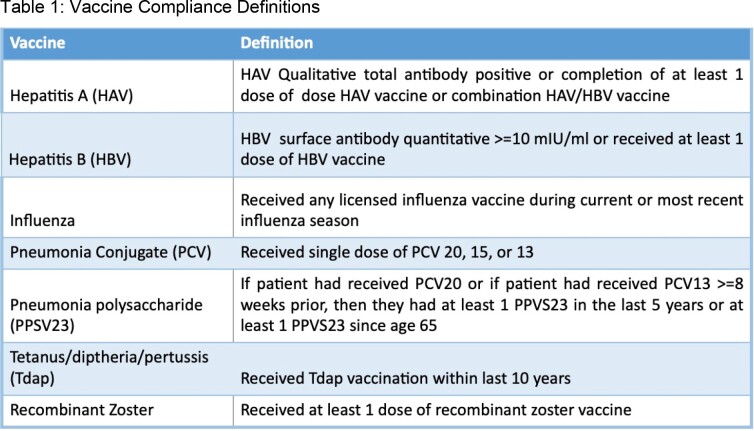

**Results:**

We included 59 patients in the pre- and 61 patients in the post-ID group(Table 2). By the end of the observation period, in those patients evaluated by ID, 30(49%) patients had undergone LT, 10 (16%) were active on waiting list,18 (30%) were not yet listed, and 4 (12.5%) were declined for LT. Among those who underwent pre-transplant ID evaluation, 53 (85.5%) patients were seen via telemedicine. Adherence was significantly higher across all vaccines among the patients evaluated by ID; the biggest change was seen in HBV, Tdap, and Zoster (Figure 1).

**Figure:**
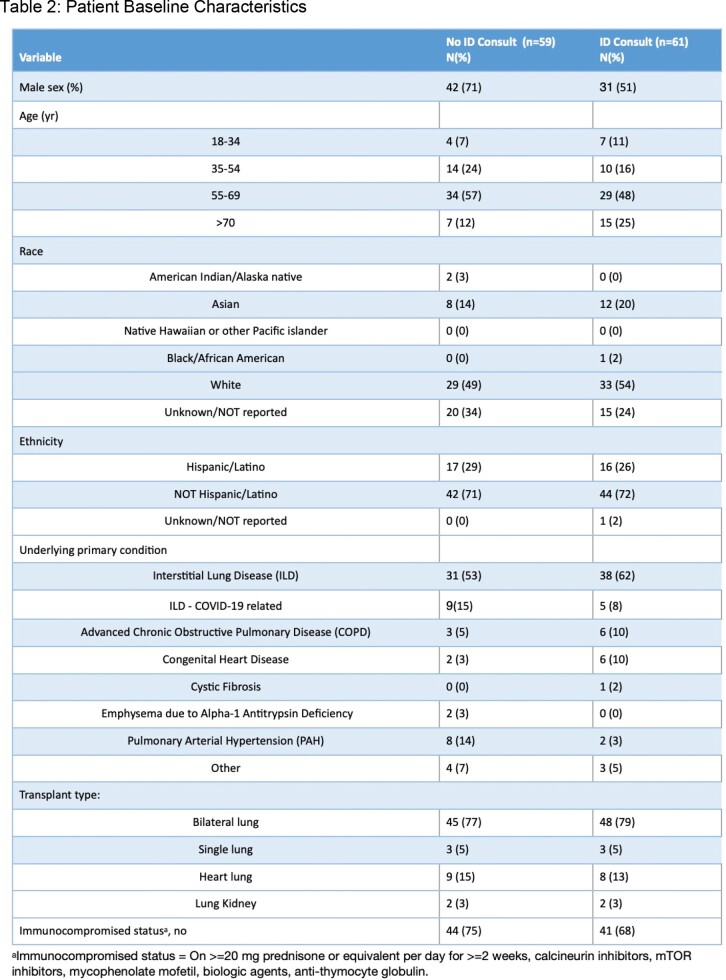

**Conclusion:**

ID evaluation of patients being evaluated for LT significantly improved vaccination rates. Pre-transplant ID evaluation provides the added value of a focused discussion of infection risk and can help to avoid morbidity and mortality post-transplant.

**Disclosures:**

**All Authors**: No reported disclosures

